# CAPS1 is involved in hippocampal synaptic plasticity and hippocampus-associated learning

**DOI:** 10.1038/s41598-021-88009-w

**Published:** 2021-04-21

**Authors:** Chiaki Ishii, Natsumi Shibano, Mio Yamazaki, Tomoki Arima, Yuna Kato, Yuki Ishii, Yo Shinoda, Yugo Fukazawa, Tetsushi Sadakata, Yoshitake Sano, Teiichi Furuichi

**Affiliations:** 1 Department of Applied Biological Science, Faculty of Science and Technology, Tokyo University of Science, 2641 Yamazaki, Noda, Chiba 278-8510 Japan; 2 Department of Environmental Health, School of Pharmacy, Tokyo University of Pharmacy and Life Sciences, 1432-1 Horinouchi, Hachioji, Tokyo 192-0392 Japan; 3 Department of Brain Structure and Function, Faculty of Medical Sciences, University of Fukui, Yoshida-gun, Fukui 910-1193 Japan; 4 Education and Research Support Center, Gunma University Graduate School of Medicine, Maebashi, Gunma 371-8511 Japan

**Keywords:** Cellular neuroscience, Synaptic transmission

## Abstract

Calcium-dependent activator protein for secretion 1 (CAPS1) is a key molecule in vesicular exocytosis, probably in the priming step. However, CAPS1’s role in synaptic plasticity and brain function is elusive. Herein, we showed that synaptic plasticity and learning behavior were impaired in forebrain and/or hippocampus-specific *Caps1* conditional knockout (cKO) mice by means of molecular, physiological, and behavioral analyses. Neonatal *Caps1* cKO mice showed a decrease in the number of docked vesicles in the hippocampal CA3 region, with no detectable changes in the distribution of other major exocytosis-related molecules. Additionally, long-term potentiation (LTP) was partially and severely impaired in the CA1 and CA3 regions, respectively. CA1 LTP was reinforced by repeated high-frequency stimuli, whereas CA3 LTP was completely abolished. Accordingly, hippocampus-associated learning was severely impaired in adeno-associated virus (AAV) infection-mediated postnatal *Caps1* cKO mice. Collectively, our findings suggest that CAPS1 is a key protein involved in the cellular mechanisms underlying hippocampal synaptic release and plasticity, which is crucial for hippocampus-associated learning.

## Introduction

In the central nervous system (CNS), a large amount of neural information is processed at a vast number of synapses. While it is widely accepted that synaptic vesicle (SV) exocytosis is executed by the core protein complex SNARE, which is composed of syntaxin-1, SNAP25, and synaptobrevin-2/VAMP2^1,2^, the fine tuning of the underlying mechanism remains unclear. The process of SV exocytosis is conveniently divided into three sequential steps, i.e., docking, priming, and fusion, although there appear to be substeps and/or no distinct boundaries among them^2,3^. In the priming step, several proteins are recruited to control the assembly of the α-helix bundle of SNARE proteins^4,5^, which is important for accomplishing the subsequent fusion step in a Ca^2+^-dependent manner.

Recent studies have shown that calcium-dependent activator protein for secretion 1 (CAPS1) is involved in the priming step of SV exocytosis^6^. This protein was originally identified as a priming factor in neuroendocrine cells for dense-core vesicle (DCV) release, rather than SV release^7,8^. Studies using *Caenorhabditis elegans* demonstrated that CAPS1 is exclusively involved in DCV release^9^. In addition, it was shown that CAPS2, a mammalian homolog of CAPS1, primarily contributes to the DCV-mediated release of neuropeptides and neurotrophins (including the brain-derived neurotrophic factor)^10–12^, but not to excitatory synaptic transmission in the mouse hippocampus^12^. However, Jockusch et al. showed that CAPS1 is essential for excitatory synaptic transmission in mouse hippocampal cultured neurons^13^, which raises the question about the regulatory roles of CAPS1 in the synapses of the mammalian CNS. Moreover, we previously reported the significance of CAPS1 in basal synaptic transmission in the CA1 region of the hippocampus because of a decrease in readily releasable SVs by CAPS1 deficiency^14^. Either Munc13-1 or Munc13-2 (or both) appear to be essential for the priming step of SVs and are involved in synaptic transmission and plasticity in the hippocampus^15^. It is also worth noting that there is functional nonredundancy between CAPS1 and Munc13-1 in the hippocampal autaptic culture system^16^. Thus, it is important to clarify the involvement of CAPS1 in presynaptic exocytosis and synaptic plasticity in neural circuits in vivo, thereby leading to higher-order brain functions, including learning behaviors. However, the functional significance of CAPS1 at the level of brain function and disease has not yet been investigated.

Taken together, these insights led us to attempt to uncover the role of CAPS1 at the levels of the hippocampal circuit and hippocampus-associated learning behavior using Cre/loxP-mediated neonatal *Caps1* conditional KO (cKO) mice (referred to as “cKO” herein after) and adeno-associated virus (AAV) infection-mediated postnatal hippocampus-specific *Caps1* cKO (referred to as “HPC-cKO” herein after) because conventional *Caps1* null mice die soon after birth^17^. Our study provides the first evidence of the importance of CAPS1 in the long-term potentiation (LTP) of two different synaptic regions within the hippocampal circuit, thereby demonstrating its essential role in hippocampus-associated learning and memory.

## Materials and methods

### Animals

All experimental protocols were evaluated and approved by the Regulation for Animal Research at the Tokyo University of Science, and were performed in compliance with the ARRIVE guidelines. All experiments were conducted in accordance with the Regulations for Animal Research at the Tokyo University of Science (approval reference no.: N18003/4, N19004/5, N20004/5). The forebrain-specific *Caps1* cKO mouse line^18^ was used in this study. Briefly, *Caps1*^*flox/flox*^ C57BL/6 female mice were crossed with *Caps1*^*flox/wt*^* Emx1*^*cre/wt*^ C57BL/6 male mice, producing *Caps1*^*flox/flox*^* Emx1*^*wt/wt*^ for control and *Caps1*^*flox/flox*^* Emx1*^*cre/wt*^ for *Caps1* cKO offspring. Male and female mice aged 2–4 months were used for electrophysiological experiments. Male and female mice aged 3–6 months were used for the biochemical experiments and behavioral tests.

### Immunohistochemistry

Immunohistochemistry was performed in accordance with previously described methods^19^. Briefly, free-floating sections (30 µm) were incubated with 0.2% (v/v) Triton X-100 in PBS and then blocked with 5% (v/v) donkey serum (Cat. S30-100ML, Chemicon), and 0.2% (v/v) Triton X-100 in PBS. The sections were incubated with primary antibodies against CAPS1 (developed in-house, 1:2000^18^), Syntaxin1 (110 011; Synaptic Systems; 1:2,000), SNAP25 (S5187; Sternberger Monoclonals Inc.; 1:2,000), VAMP2 (104 211; Synaptic Systems; 1:2000), or vGluT1 (135,303; Synaptic Systems; 1:1000) at 4 °C over two nights. The samples were then incubated with Alexa Fluor 488 goat anti-mouse IgG (A21202) or Alexa Fluor 555 goat anti-rabbit IgG (A-21428) secondary antibody (Thermo Fisher). The sections were imaged using a confocal microscope (FV1000 IX81; Olympus) equipped with a 100 × oil immersion UPlanSApo objective (NA 1.40; Olympus). Images were acquired using FV-10 ASW software (OLYMPUS) and analyzed using the ImageJ software. Immunofluorescence signals beyond arbitrary thresholds were selected for puncta analysis.

### Subcellular fractionation

Subcellular fractionation was conducted in accordance with previously described methods^20^, with slight modifications. Briefly, hippocampi were removed from whole brains and placed into 500 µL of high-sucrose homogenate buffer (320 mM sucrose and 4 mM HEPES–NaOH, pH 7.4) supplemented with complete ULTRA Tablets Mini EASYpack (Roche) and PhosSTOP (Roche). The hippocampi were then homogenized using a hand homogenizer (Fisher Scientific) equipped with a disposable pestle (Fisher Scientific). All processes were performed on ice. The homogenized hippocampal solution was then centrifuged (800 × *g*, 15 min, 4 °C) to remove the perikaryon. Finally, the supernatant was centrifuged again (9,200 × *g*, 15 min, 4 °C) to obtain the P2 fraction as a pellet that included the synaptosomes.

### Western blotting

Subcellular P2 fractions were solubilized in SDS sample buffer (50 mM Tris, 4% SDS, 0.01% Serva Blue G, 12% glycerol, and 2% β-mercaptoethanol) for 5 min at 100 °C. The eluted proteins were then separated in 12% (for syntaxin1, SNAP25, and VAMP2) or 6% (for CAPS1, Munc13, and Munc18-1) SDS–polyacrylamide gels and transferred to PVDF membranes. To detect the proteins of interest, anti-syntaxin1 (110 011; Synaptic Systems; 1:6,000), anti-SNAP25 (S5187; Sternberger Monoclonals Inc.; 1:6,000), anti-VAMP2 (104 211; Synaptic Systems; 1:6000), anti-Munc18-1 (ab3451; Abcam; 1:8000), and anti-CAPS1 (developed in-house, 1:6000^18^) antibodies were used as primary antibodies, with peroxidase-labeled goat anti-mouse or rabbit IgG antibodies (074-1806 or 074-1506, respectively; KPL; 1:100,000) used as secondary antibodies. The chemiluminescent reaction was initiated by applying Immunobilon Western Chemiluminescent HRP Substrate (Millipore). After the detection of the proteins of interest, the chemiluminescent membranes were incubated in 15% H_2_O_2_/PBS for 30 min at room temperature, to inactivate peroxidase-labeled secondary antibodies, followed by the detection of β-actin as an internal control (017-24573; Wako; 1:10,000). The chemiluminescent reaction was detected using an LAS-4000 mini image analyzer (FUJIFILM), and images were analyzed using ImageJ software (NIH) (Image J for Mac OS X; ImageJ bundled with Java1.8.0_172 (https://imagej.nih.gov/ij/download.html).

### Electron microscopy

Electron microscopic analysis was performed as described previously^14,21^. Briefly, mice were anesthetized with CO_2_ gas and perfused with PBS, followed by 25 mL of a mixture of 2.5% glutaraldehyde and 2% paraformaldehyde in 0.1 M PB. The brains were removed and post-fixed in the same fixative at 4 °C overnight. The fixed brains were cut into coronal sections (300 µm thick) using a microslicer (VT1000S;Leica). The slices were placed in a cold 1%OsO4 solution for 1 h. The slices were dehydrated with a graded ethanol series and mounted with epoxy resin (EPON812; TAAB Laboratories). Next, 70 nm serial sections were prepared using an ultramicrotome (EMUC6; Leica). Images of the CA3 stratum lucidum were acquired using a transmission electron microscope (1200EX; Jeol) at 80 kV. The images were analyzed using Photoshop (Adobe) and ImageJ (NIH) software.

### Preparation of hippocampal acute slices

Acute hippocampal slices were prepared as described previously^12^. Briefly, mice were decapitated under deep anesthesia with CO_2_, and whole brains were rapidly removed into chilled high-sucrose Ringer’s solution (234 mM sucrose, 2.5 mM KCl, 1.25 mM NaH_2_PO_4_, 10 mM MgSO_4_, 0.5 mM CaCl_2_, 26 mM NaHCO_3_, and 11 mM d-glucose; pH 7.5). Hippocampi were transversely cut into 400-µm-thick slices in the same chilled high-sucrose Ringer’s solution using a linear slicer (Dosaka, Kyoto, Japan), followed by recovery in ACSF (125 mM NaCl, 2.5 mM KCl, 1.25 mM NaH_2_PO_4_, 1 mM MgCl_2_, 2 mM CaCl_2_, 26 mM NaHCO_3_, and 11 mM d-glucose; pH 7.5) at room temperature for at least 2 h. All solutions were constantly bubbled with 95% O_2_/5% CO_2_.

### Electrophysiology

Electrophysiological field recordings were performed as described previously^12^. All recordings were performed in ACSF at 26 °C. ACSF was exchanged at a rate of 1 mL/min. A bipolar tungsten-stimulating electrode (WPI) was used as the stimulation electrode, and an Ag/AgCl recording electrode was placed in a glass pipette (Harvard Apparatus) filled with ACSF. To record the CA1 region, the stimulation electrode was placed on the stratum radiatum of the CA1, and fEPSPs were recorded in the absence of picrotoxin. To record the CA3 region, the stimulation electrode was placed on the hilus of the DG region, and fEPSPs were recorded from the stratum lucidum of the CA3. To record LTP from the CA3 region (but not for the recording of basal transmission and PPF), ACSF containing 100 μM picrotoxin (Sigma) was perfused into the recording chamber because trace levels of synaptic transmission hamper the discrimination of signals from the noise without blocking GABA-A receptors. DCG4 (Nacalai) was applied after the completion of all recordings in the CA3 region (10 µM) to confirm that few responses from the commissural/associational pathway were observed^22^. TBS-induced LTP was elicited by four trains, with 10 s intervals between each train. Each train consisted of five bursts separated by 200 ms and included four pulses delivered at 100 Hz, which were applied at 40–50% of the maximal stimulus intensity. Electrical signals were amplified using a MultiClamp 700A instrument (Molecular Devices), digitized at 10 kHz, and filtered at 2 kHz using a Digidata 1440 system with the pCLAMP10 software (Molecular Devices). Amplitude and slope were analyzed for all fEPSPs. For slope data, fEPSPs were analyzed over 30–70% of the rising slope. The results were depicted at 30–70% of the rising slopes for all data, with the exception of the LTP data from the CA3 region because of the very small waveforms recorded in *Caps1* cKO slices. The fEPSPs from the CA3 region were analyzed over 0–100% of the rising slope only for LTP data. This enabled a detailed analysis of this small fEPSP waveform phenotype in *Caps1* cKO mice during long recordings.

### Contextual fear-conditioning test

Mice were housed individually for at least 5 days before behavioral testing. All training and testing experiments were performed during the light cycle. A fear-conditioning shock chamber consisting of a Plexiglass front and gray sides/back walls, with 26 stainless steel grids on a square-shaped floor (18 w × 17 D × 40 H cm) (Muromachi) was used. Each mouse was placed inside the conditioning chamber and allowed to explore for 3 min. The mice then received three shocks (0.4 mA for 2 s) with a 60 s interstimulus interval. The mice were left in the conditioning chamber for 1 min after the last shock. After the conditioning session, the contextual test was administered over 24 h for the LTM test or 30 min for the STM test. The mice were then returned to the same conditioning chamber, and their behavior was monitored for 5 min. The freezing behavior of the mice was monitored using a video camera and processed using a DVTrack video tracking system (Muromachi).

### Auditory fear-conditioning test

A fear-conditioning shock chamber consisting of a white square chamber (18 w × 17 D × 40 H cm) with 26 stainless-steel grids on the floor (Muromachi) was used. Each mouse was placed inside the conditioning chamber and allowed to explore for 3 min. The mice then received a 3-tone-shock paired with a 2 min interstimulus interval. Each tone-shock pairing comprised an auditory cue (80 dB of a 4 kHz tone, 30 s long) that coterminated with an electric footshock (0.4 mA, 2 s long). The mice were left in the conditioning chamber for 1 min after the last tone-shock pairing. The retrieval test was performed 2 days after conditioning. For the retrieval test, another testing chamber with very different properties was used. This chamber consisted of a white regular triangular chamber (18 × 40 cm) with a floor covered with white Kim towels. The mice were placed inside the chamber and allowed to explore for 2 min. The same tone used in the conditioning session (80 dB of a 4 kHz tone, 30 s long) was administered for 2 min. The freezing behavior of the mice was monitored using a video camera and processed using a DVTrack video tracking system (Muromachi).

### Context-discrimination task

Mice were housed individually for at least 5 days before behavioral testing. All training and testing experiments were performed during the light cycle. The fear-conditioning shock chamber (chamber A) consisted of a Plexiglass front and gray sides/back walls, with 26 stainless steel grids on a square-shaped floor (18 w × 17 D × 40 H cm) (Muromachi). A non-fear-conditioning shock chamber (chamber B) consisted of a Plexiglass front covered with a paper towel, two beige side walls, and a triangular floor (18 sides × 40 H cm) without grids (Muromachi). The paper towel containing 100 × diluted detergent (Simple Green) was hidden under the triangle-shaped floor to discriminate the odor cue from that of chamber A. For the conditioned trials, each mouse was placed inside chamber A and allowed to explore for 45 s. The mice then received a single electrical shock (0.4 mA for 2 s) and were left in the conditioning chamber for 15 s after the shock. One day after the conditioning trial, each mouse was placed inside chamber B and allowed to explore for 60 s. These two trials were performed alternately (see the experimental scheme in Fig. S4A). The freezing behavior of the mice was monitored using a video camera and processed using a DVTrack video tracking system (Muromachi).

### Adeno-associated virus injection

The *AAV8-CaMKIIα:eGFP* (10^12^ genome copies/mL) and *AAV8-CaMKIIα:eGFP-Cre* (10^12^ genome copies/mL) were generated from the University of North Carolina at Chapel Hill Vector Core. *Caps1*^*flox/flox*^ mice were mounted in a stereotaxic apparatus and anesthetized using pentobarbital (80 mg/kg) with an analgesic (5 mg/kg, Carprofen). One microliter of virus solution was infused bilaterally using a Hamilton syringe through a glass micropipette at the following coordinates (relative to bregma [mm] ): anterior–posterior [AP], − 2.0 mm from bregma; medial–lateral [ML], ± 2.0 mm) from the bregma; dorsal–ventral [DV], − 2.0 mm from the surface of the skull at bregma; and AP, − 3.2 mm; ML, ± 3.5 mm; DV, 3.6 mm) taken from the mouse brain atlas^23^ at a rate of 0.1 mL/min. A glass capillary was left in place for an additional 5 min. Behavioral tests were performed 4 weeks after surgery to allow for animal recovery and sufficient expression of genes.

### Statistical analyses

All data are reported as the mean ± SEM. Differences between data sets were assessed using two-tailed Student’s *t*-test (for unpaired data), paired *t*-test (for paired data), repeated two-way ANOVA (for data including three or more groups with two independent variables), or the Tukey–Kramer test (for data including three or more groups with a single variable). All data were collected and analyzed using a double-blinded approach. The validity of statistical power was confirmed by *post-hoc* statistical power analyses (Supplementary Table S1).

### Ethics statement

The animal study was reviewed and approved by the Institutional Animal Care and Use Committee of the Tokyo University of Science (approval reference no.: N18003/4, N19004/5, N20004/5).

## Results

### The expression levels and distribution patterns of major presynaptic components in SV exocytosis are not perturbed by the absence of CAPS1

**Figure 1:**
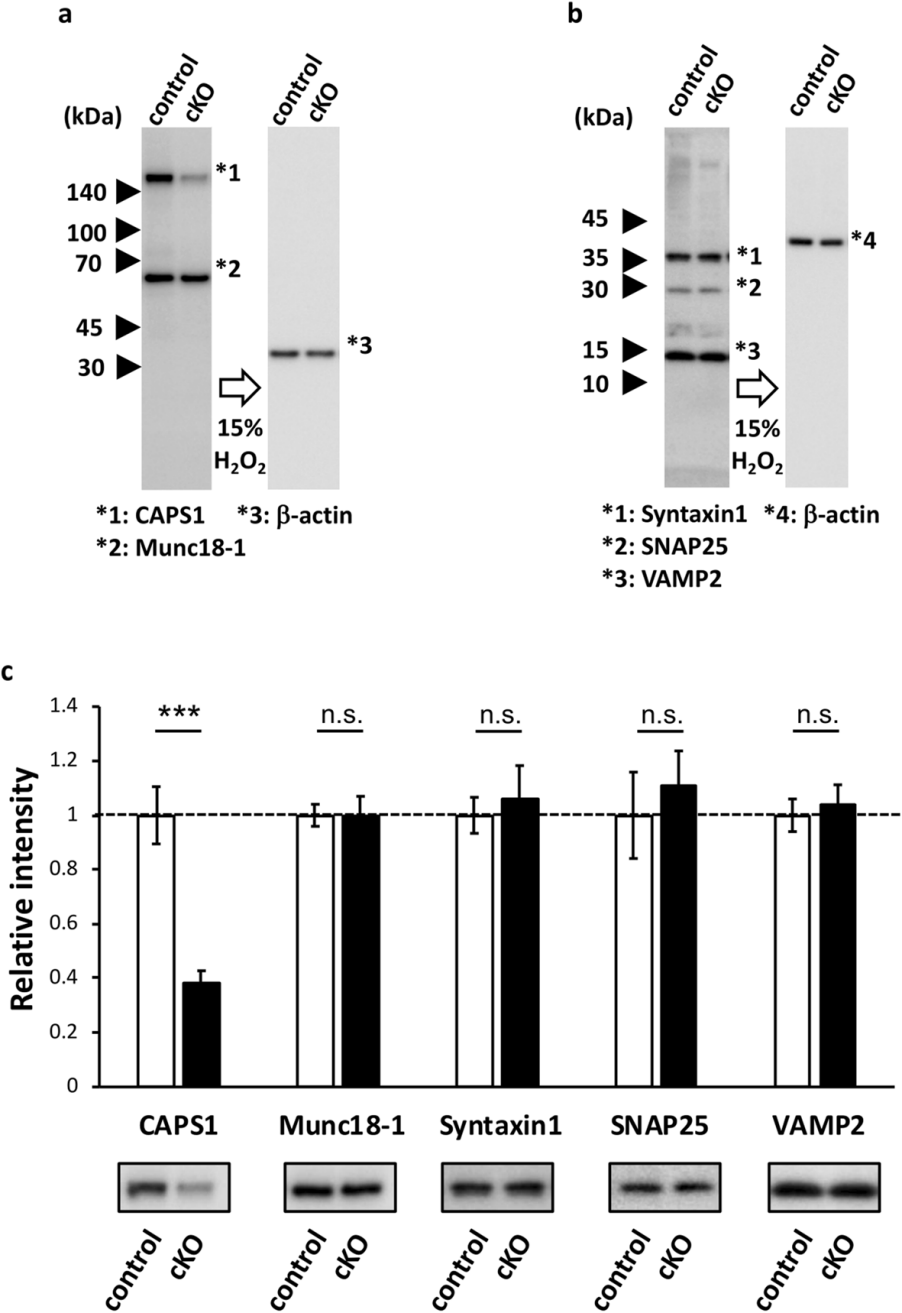
Expression levels of presynaptic proteins that contribute to SV release in the *Caps1* cKO hippocampus. (**a**) Representative Western blot images of CAPS1 and Munc18-1. Synaptosomal fractions were prepared from hippocampal tissue homogenates (1 µg of total protein per lane). CAPS1, and Munc18-1 were detected on the same membrane. β-actin was used as an internal control to normalize the expression levels of each protein after treatment with 15% H_2_O_2_. (**b**) Representative Western blot images of syntaxin-1, SNA25, and synaptobrevin/VAMP2. Synaptosomal fractions were prepared from hippocampal tissue homogenates (1 µg of total protein per lane). Syntaxin-1, SNA25, and synaptobrevin/VAMP2 were detected on the same membrane. β-actin was used as an internal control to normalize the expression levels of each protein after treatment with 15% H_2_O_2_. (**d**) Expression levels of presynaptic proteins in control and *Caps1* cKO hippocampal synapses. An image of a representative band is depicted below the corresponding bar graph. The expression level of each protein in *Caps1* cKO hippocampi was standardized to that of control hippocampi. The blots in Fig. 1 were cropped from the same parts of the same gels as shown in Supplementary Figure S1. (**a**,**b**) were from Figure S1b and (**a**), respectively. Similarly, the blots in (**d**) were also derived from the corresponding blots shown in Supplementary Figure S1. Control, n = 6 animals; *Caps1* cKO, n = 6 animals. *P* = 0.974, 0.700, 0.601 and 0.682 for Munc18, Syntaxin1, Smap25 and VAMP2, respectively, Student’s *t*-test.

To examine whether this physiological impairment was solely due to the lack of CAPS1 or secondary effects by other molecules, we confirmed the expression levels and cellular distribution patterns of six exocytosis-related proteins. To this end, we performed western blotting on synaptosomal fractions from hippocampal tissue homogenates, probing for CAPS1, Munc18-1, Syntaxin1, SNAP25, and Synaptobrevin/VAMP2 (Fig. 1a,b). Our results indicated that the expression of these presynaptic proteins did not change between *Caps1* cKO and control mice, except for a reduction in CAPS1 expression in cKO mice (Fig. 1c). The membranes used in this study are shown in Supplementary Fig S1.

**Figure 2:**
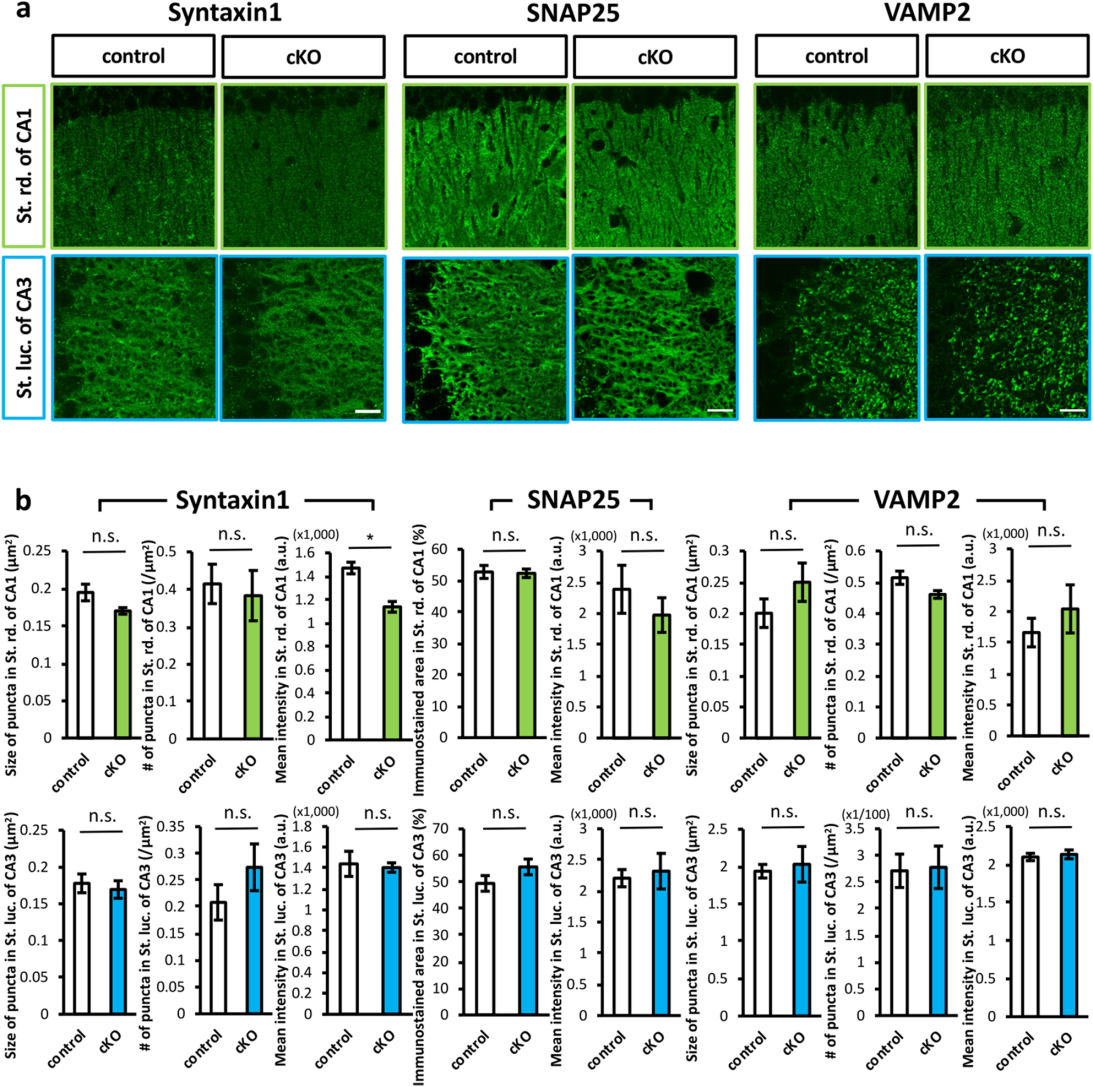
The expression patterns of SNARE proteins in *Caps1* cKO hippocampus. (**a**) Localization patterns of presynaptic proteins in the stratum radiatum of CA1 region and the stratum lucidum of CA3 region. Immunohistochemical staining for Syntaxin1, SNAP25, and Synaptobrevin/VAMP2 were performed at least in duplicate and their representative images are shown. Upper: Stratum radiatum of CA1. Lower: Stratum lucidum of CA3. Left: control slices. Right: *Caps1* cKO slices. Scale bars: 20 µm. (**b**) Quantitative analyses for the histological expression patterns of SNARE proteins. Puncta size of Syntaxin1 in CA1: control, n = 5 animals; *Caps1* cKO, n = 5 animals. *P* = 0.073, Student’s *t*-test. Puncta size of Syntaxin1 in CA3: control, n = 5 animals; *Caps1* cKO, n = 5 animals. *P* = 0.646, Student’s *t*-test. Puncta density of Syntaxin1 in CA1: control, n = 5 animals; *Caps1* cKO, n = 5 animals. *P* = 0.722, Student’s *t*-test. Puncta density of Syntaxin1 in CA3: control, n = 5 animals; *Caps1* cKO, n = 5 animals. *P* = 0.266, Student’s *t*-test. Mean intensity of Syntaxin1 in CA1: control, n = 5 animals; *Caps1* cKO, n = 5 animals. *P* = 0.001, Student’s *t*-test. Mean intensity of Syntaxin1 in CA3: control, n = 5 animals; *Caps1* cKO, n = 5 animals. *P* = 0.789, Student’s *t*-test. Area of SNAP25 in CA1: control, n = 5 animals; *Caps1* cKO, n = 5 animals. *P* = 0.866, Student’s *t*-test. Area of SNAP25 in CA3: control, n = 5 animals; *Caps1* cKO, n = 5 animals. *P* = 0.175, Student’s *t*-test. Mean intensity of SNAP25 in CA1: control, n = 5 animals; *Caps1* cKO, n = 5 animals. *P* = 0.409, Student’s *t*-test. Mean intensity of SNAP25 in CA3: control, n = 5 animals; *Caps1* cKO, n = 5 animals. *P* = 0.732, Student’s *t*-test. Puncta size of VAMP2 in CA1: control, n = 5 animals; *Caps1* cKO, n = 5 animals. *P* = 0.232, Student’s *t*-test. Puncta size of VAMP2 in CA3: control, n = 5 animals; *Caps1* cKO, n = 5 animals. *P* = 0.726, Student’s *t*-test. Puncta density of VAMP2 in CA1: control, n = 5 animals; *Caps1* cKO, n = 5 animals. *P* = 0.061, Student’s *t*-test. Puncta density of VAMP2 in CA3: control, n = 5 animals; *Caps1* cKO, n = 5 animals. *P* = 0.899, Student’s *t*-test. Mean intensity of VAMP2 in CA1: control, n = 5 animals; *Caps1* cKO, n = 5 animals. *P* = 0.423, Student’s *t*-test. Mean intensity of VAMP2 in CA3: control, n = 5 animals; *Caps1* cKO, n = 5 animals. *P* = 0.654, Student’s *t*-test.

We subsequently performed immunohistochemical staining to analyze the synaptic localization of some of the SNARE proteins in the stratum radiatum of the CA1 region and the stratum lucidum of the CA3 region. The overall immunostaining patterns for SNARE proteins in the two regions appeared to be similar between the two genotypes, with the exception that the mean intensity of Syntaxin1 in the stratum radiatum of CA1 was significantly decreased in *Caps1* cKO (Fig. 2a,b), indicating that the cellular expression levels of SNARE proteins were not severely affected by loss of CAPS1. However, subtle changes at each synapse level remain elusive, because our previous electron microscopy study showed aberrant distribution and accumulation of SVs in the CA1 region of cKO mice^14^.

In addition, the distribution pattern of putative excitatory synapses was evaluated (Supplementary Fig. S2). All of these biochemical results indicated that the absence of CAPS1 in the matured hippocampal circuit did not affect the expression level of exocytotic machinery or presynaptic morphology.

### *Caps1* deficiency causes a decrease in the docked vesicles and an increase in the distal vesicles in hippocampal presynapses

**Figure 3:**
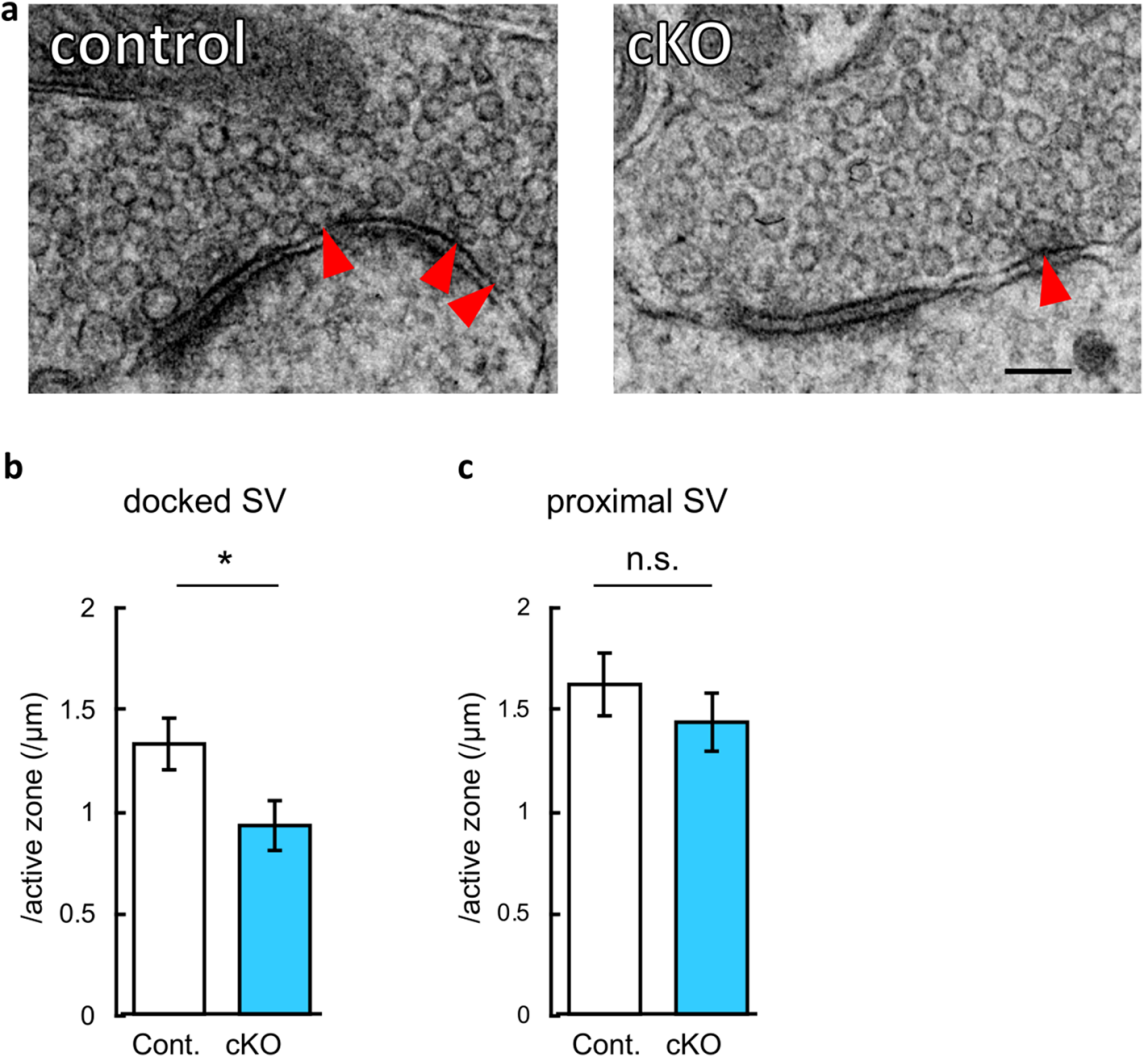
Electron microscopic analyses of the stratum lucidum of the CA3 region in the *Caps1* cKO hippocampus. (**a**) Representative electron microscopic images of mossy fiber terminals. The red arrowheads indicate docked SVs on the active zones; Scale bar, 100 nm. (**b**) Number of SVs docked on the active zones in a mossy fiber terminal. Control, n = 71 active zones from 3 animals; *Casp1* cKO, n = 75 active zones from 3 animals; *P* = 0.028, Student’s *t*-test. (**c**) Number of SVs in the proximity of the active zones (within 50 nm, but not docked) in a mossy fiber terminal. Control, n = 71 active zones from 3 animals; *Casp1* cKO, n = 75 active zones from 3 animals; *P* = 0.320, Student’s *t*-test.

Our previous electron microscopic study showed a marked reduction in the number of docked SVs, but an accumulation of total SVs was observed in the CA1 presynapses of *Caps1* cKO mice^14^. Subsequently, we analyzed the presynaptic distribution of SVs in the CA3 region. Consistent with the CA1 region, docked SVs in the active zones were reduced, although proximal SVs that were distributed within 50 nm from the active zones did not change (Fig. 3a–c). Thus, these results suggest that CAPS1 is required for SVs to be ready for exocytosis, whereas the deficit of CAPS1 causes abnormal SV distribution in the presynapses of the hippocampus.

### LTP is perturbed in the CA1 region and severely impaired in the CA3 region, in *Caps1* cKO acute hippocampal slices

**Figure 4:**
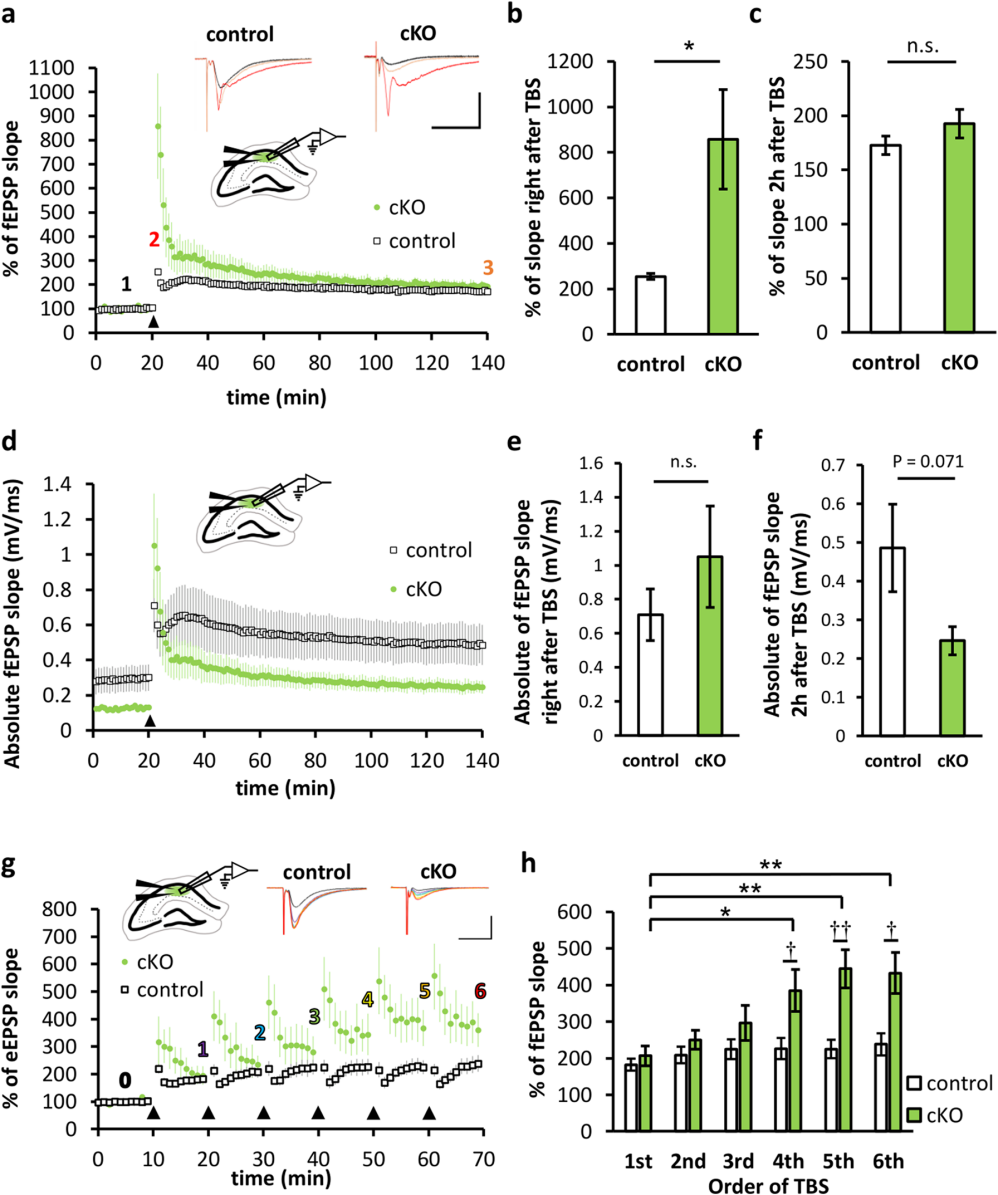
LTP pattern in the CA1 region of *Caps1* cKO hippocampal slices. (**a**) Changes in fEPSP slope during LTP induced by TBS in the CA1 region (ratio to the baseline). TBS was applied at the 20 min point (indicated by the black triangle). Representative waveforms recorded at the baseline (black, 1), immediately after TBS (red, 2), and 2 h after TBS (orange, 3) are shown in the top panel (vertical, 2 mV; horizontal, 10 ms). (**b**) Potentiated fEPSP slope levels recorded immediately after TBS in the CA1 region. Control, n = 6 animals; *Casp1* cKO, n = 5 animals; *P* = 0.014, Student’s *t*-test. (**c**) Potentiated fEPSP slope levels at 2 h after TBS in the CA1 region. Control, n = 6 animals; *Casp1* cKO, n = 5 animals; *P* = 0.222, Student’s *t*-test. (**d**) Changes in absolute fEPSP slope values during LTP induced by TBS in the CA1 region. TBS was applied at the 20 min point (indicated by the black triangle). (**e**) Absolute value of the fEPSP slope immediately after TBS. Control, n = 6 animals; *Casp1* cKO, n = 5 animals; *P* = 0.314, Student’s *t*-test. (**f**) Absolute value of the fEPSP slope at 2 h after TBS. Control, n = 6 animals; *Casp1* cKO, n = 5 animals; *P* = 0.071, Student’s *t*-test. (**g**) Changes in fEPSP slope during LTP induced by repeated TBS at 10 min intervals. TBS was applied at the 10, 20, 30, 40, 50, and 60 min points (indicated by black triangles). The representative waveforms recorded at the baseline (black, 0) and with sustained levels induced by the 1st TBS (purple, 1), 2nd TBS (light blue, 3), 3rd TBS (light green, 3), 4th TBS (yellow, 4), 5th TBS (orange, 5), and 6th TBS (red, 6) are shown in the top panel. Scale bars: vertical, 1 mV; horizontal, 20 ms. (H) Sustained potentiation ratios recorded 10 min after the 1st to the 6th TBS. *P < 0.05, **P < 0.01; 1st vs. n-th, †*P* < 0.05; control vs. *Caps1* cKO, Student’s *t*-tests. Number (n) of animals used for statistical analyses in this experiment: n = 5 for control and n = 5 for cKO.

Previously, we demonstrated that basal synaptic transmission was severely impaired in the CA1 region of *Caps1* cKO acute hippocampal slices^14^. Therefore, we next investigated LTP at these synapses using field recordings from acute hippocampal slices. We recorded the field excitatory postsynaptic potential (fEPSP) in the stratum radiatum of the CA1 region. The slope of fEPSPs in *Caps1* cKO slices was significantly augmented immediately after the application of theta-burst stimulation (TBS), followed by a rapid decrease toward the baseline level (Fig. 4a). The augmented level observed immediately after TBS was significantly greater in *Caps1* cKO slices than that in control slices (Fig. 4b). This high level of fEPSP detected in *Caps1* cKO slices was not observed 2 h after TBS, whereas the potentiation level was very similar to that of the control slices (Fig. 4c). However, the absolute value of the fEPSP slope in *Caps1* cKO slices tended to be smaller (Fig. 4d–f), probably because of the suppressed balsa synaptic transmission level. Our results demonstrated that CA1 LTP can be induced and maintained in the absence of CAPS1, although the absolute transmission level remains low under these conditions. Moreover, repeated TBS administered at 10 min intervals yielded a gradual enhancement in the potentiation levels in the CA1 region of *Caps1* cKO slices in accordance with the number of TBS applications (Fig. 4g,h), indicating that CAPS1-deficient synapses exhibit a hidden capacity to potentiate synaptic transmission in the CA1 region.

**Figure 5:**
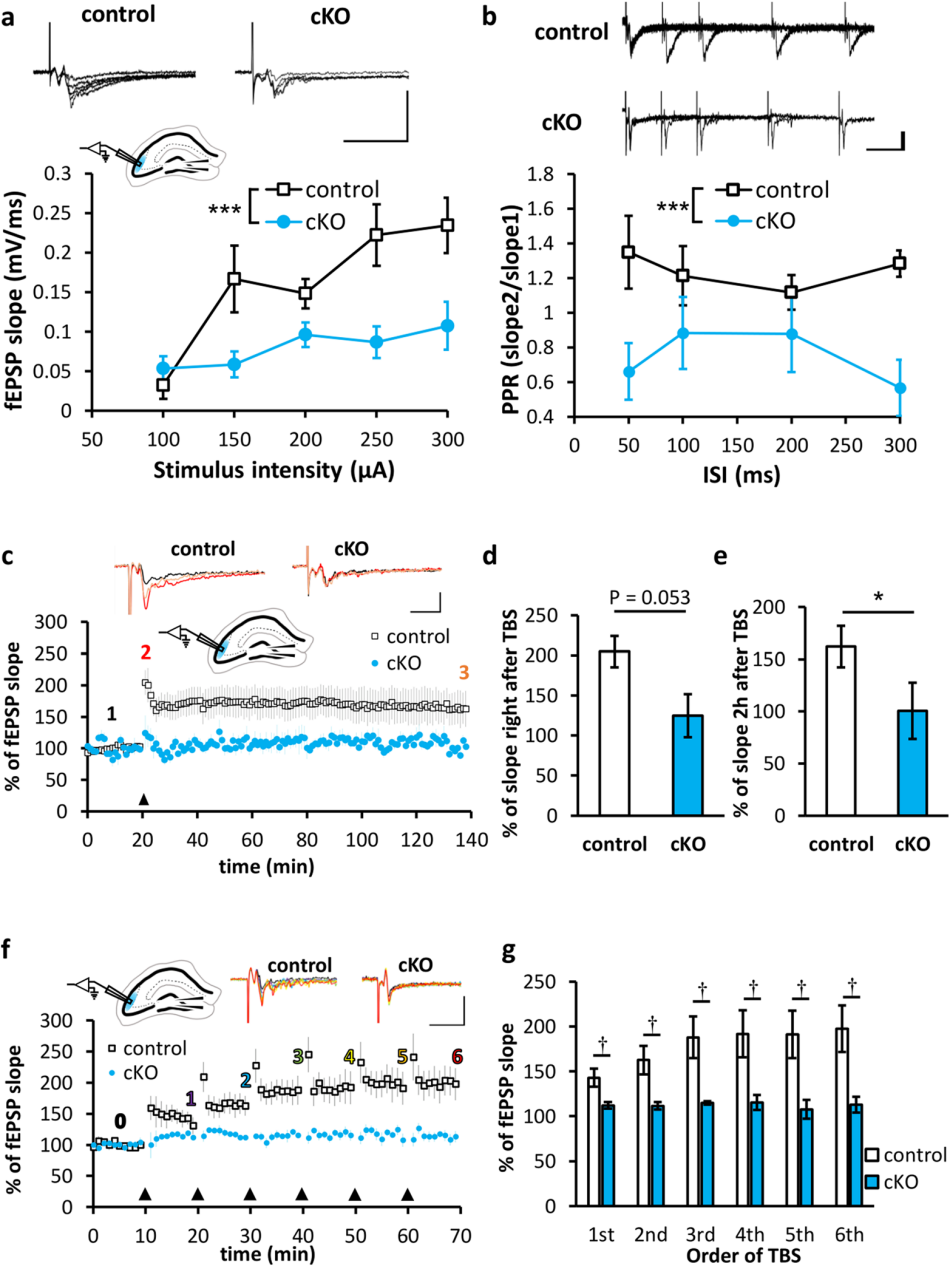
LTP pattern in the CA3 region of *Caps1* cKO hippocampal slices. (**a**) Input–output curve of synaptic transmission in the CA3 region. Representative waveforms are depicted in the upper panel (vertical, 500 µV; horizontal, 20 ms). The graph in the lower panel shows the fEPSP slope of electrical stimulation intensity. Control, n = 5 animals; *Casp1* cKO, n = 7 animals; ***P < 0.001, repeated two-way ANOVA (Genotype; F_(1, 10)_ = 24.88, *P* < 0.001). (**b**) Paired-pulse ratio (PPR) recorded from the CA3 region. Representative waveforms are depicted in the upper panel (vertical, 100 µV; horizontal, 50 ms). The graph in the lower panel shows PPRs calculated by dividing the second fEPSP slope by the first fEPSP slope to interstimulus intervals. Control, n = 5 animals; *Casp1* cKO, n = 7 animals; ***P < 0.001, repeated two-way ANOVA (Genotype; F_(1, 10)_ = 24.47, *P* < 0.001)(**c**) Changes in fEPSP slope during LTP induced by TBS in the CA3 region (ratio to the baseline). TBS was applied at the 20 min point (indicated by the black triangle). Representative waveforms recorded at the baseline (black, 1), immediately after TBS (red, 2), and 2 h after TBS (orange, 3) are shown in the top panel (vertical, 200 µV; horizontal, 10 ms). (**d**) Potentiated fEPSP slope levels recorded immediately after TBS in the CA3 region. Control, n = 5 animals; *Casp1* cKO, n = 7 animals; *P* = 0.053, Student’s *t*-test. (**e**) Potentiated fEPSP slope levels at 2 h after TBS. Control, n = 5 animals; *Casp1* cKO, n = 7 animals; *P* = 0.023, Student’s *t*-test. (**f**) Changes in fEPSP slope during LTP induced by repeated TBS at 10 min intervals. TBS was applied at the 10, 20, 30, 40, 50, and 60 min points (indicated by black triangles). The representative waveforms recorded at the baseline (black, 0) and with sustained levels induced by the 1st TBS (purple, 1), 2nd TBS (light blue, 3), 3rd TBS (light green, 3), 4th TBS (yellow, 4), 5th TBS (orange, 5), and 6th TBS (red, 6) are shown in the top panel. Scale bars: vertical, 200 µV; horizontal, 20 ms. (G) Sustained potentiation ratios recorded 10 min after the 1st to the 6th TBS. *P* < 0.05; control vs. cKO, Student’s *t*-tests. Number (n) of animals used for statistical analyses in this experiment: n = 6 for control and n = 5 for cKO.

Next, we carefully applied electrical stimulation to the hilus of the dentate gyrus (DG) region of the hippocampus and recorded fEPSPs from the stratum lucidum of the CA3 region. As in the case of the CA1 region, basal transmission was suppressed (Fig. 5a), and putative readily releasable SVs (docked vesicles) were reduced (Fig. 3) in the CA3 region of *Caps1* cKO slices. Notably, the paired-pulse ratio was slightly decreased in the CA3 region of *Caps1* cKO slices (Fig. 5b), indicating that the readily releasable pool was severely reduced in this type of synapse. Because of this insight and the fact that several inhibitory neurons innervate postsynaptic CA3 pyramidal cells as well as presynaptic DG granule cells^24,25^, we added 100 μM picrotoxin, a blocker of ionotropic GABA receptors, to induce LTP in the CA3 region. In contrast to that observed in the CA1 region, the fEPSP slope remained very small after the application of TBS in the CA3 region of *Caps1* cKO slices (Fig. 5c). The fEPSP slope recorded immediately after TBS tended to be smaller in *Caps1* cKO slices than that in control slices (Fig. 5d). In addition, the potentiation level recorded 2 h after TBS was significantly different between the control and *Caps1* cKO mice (Fig. 5e). Repeated TBS administration revealed that LTP was completely abolished in the CA3 area (Fig. 5f,g). To verify the contribution of mossy fiber input to the fEPSPs evoked in the CA3 region, we administered DCG4, an agonist of the type II metabotropic glutamate receptors that are present at mossy fiber terminals but not CA3 recurrent synapses, to the recording chamber. Based on the strong inhibitory effects of DCG4 (Supplementary Fig. S3), we assumed that the CA3 fEPSPs were obtained mostly as a response to mossy fiber input from the DG region, although further investigation (such as a patch-clamp recording study) might be required to definitively evaluate this response. Taken together, our data demonstrate that CAPS1 is required not only for the long-term maintenance but also for the induction of CA3 LTP.

Collectively, these findings indicate that CAPS1 is required for the normal LTP magnitude in the CA1 region, whereas CAPS1 is essential for the initiation of CA3 LTP.

### *Caps1* cKO mice show hippocampus-dependent memory deficits

**Figure 6:**
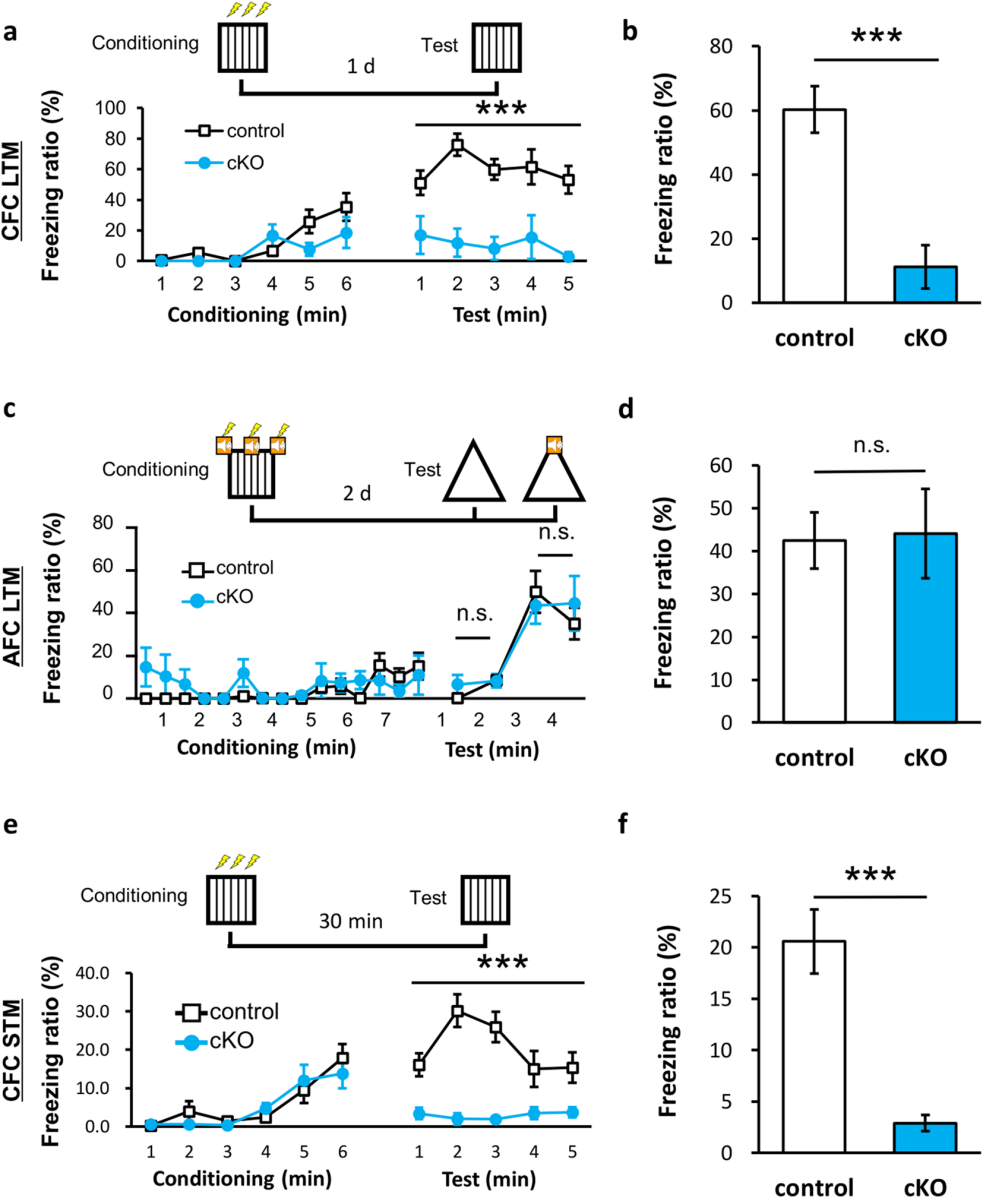
Memory deficits in *Caps1* cKO adult mice. (**a**) Changes in the freezing ratio during the CFC conditioning and LTM retrieval tests. The schematic timeline is depicted above the line graph. ***P < 0.001, repeated two-way ANOVA (Genotype; F_(1, 9)_ = 5.09, *P* = 0.505 during shock in conditioning phase (1–3 min); F_(1, 9)_ = 1.14, *P* = 0.313 during baseline in conditioning phase (4–6 min); F_(1, 9)_ = 23.87, *P* < 0.001 during retrieval test). (**b**) Mean freezing ratio during the LTM retrieval test. Control, n = 6 animals; *Caps1* cKO, n = 5 animals. ***P < 0.001, Student’s *t*-test. (**c**) Changes in the freezing ratio during AFC conditioning and LTM retrieval testing. The schematic timeline is depicted above the line graph. No significant change between the two groups occurred, repeated two-way ANOVA (Genotype; F_(1, 10)_ = 2.85, *P* = 0.122 at baseline in the conditioning phase (0.5–3 min); F_(1, 10)_ = 0.01, *P* = 0.912 during tone with shock in conditioning phase (3.5–7.5 min); F_(1, 10)_ = 0.91, *P* = 0.895 at baseline in retrieval test (1–2 min); F_(1, 10)_ = 0.02, *P* = 0.362 during tone in retrieval test (3–4 min)). (**d**) Mean freezing ratio during tone representation in the AFC-LTM test. Control, n = 7 animals; *Caps1* cKO, n = 5 animals. *P* = 0.895, Student’s *t*-test. (**e**) Changes in the freezing ratio during CFC conditioning and STM retrieval testing. The schematic timeline is depicted above the line graph. ***P < 0.001, repeated two-way ANOVA (Genotype; F_(1, 28)_ = 1.08, *P* = 0.307 during baseline in conditioning phase; F_(1, 28)_ = 0.01, *P* = 0.935 during shock in conditioning phase; F_(1, 28)_ = 18.08, *P* < 0.001 during retrieval test). (**f**) Mean freezing ratio during the CFC-STM retrieval test. Control: n = 19 animals; *Caps1* cKO: n = 11 animals. ***P < 0.001, Student’s *t*-test.

Next, we investigated whether deficits in synaptic function in *Caps1* cKO mice affect learning and memory. *Caps1* cKO mice were trained in the contextual fear-conditioning (CFC) task and were tested 1 d after training. In the long-term memory (LTM) test, *Caps1* cKO mice showed significantly decreased freezing levels compared with that in the control littermates (Fig. 6a,b). Next, an auditory fear-conditioning (AFC) test was performed. In the AFC-LTM test, the freezing ratio recorded during tone representation was indistinguishable between the two groups (Fig. 6c,d). These results suggest that the dysfunction of CFC memory formation is caused by impairment in encoding contextual information. We then tested short-term memory (STM) formation, in which mice were trained in the CFC task and tested 30 min after training. In the CFC-STM test, the freezing ratio significantly decreased in *Caps1* cKO mice compared with the control littermates (Fig. 6e,f). The freezing levels recorded during the conditioning phase of each experiment were not significantly different between the groups (Fig. 6a,c,e). Next, we tested the ability to discriminate the context of *Caps1* cKO mice. Mice were allowed to explore in context A (conditioned context) and received a weak electrical shock. On the following day, the mice were exposed to context B (unconditioned context). Next, the mice were alternately exposed to context A and B every day. As a result, *Caps1* cKO mice hardly discriminated between contexts A and B (Supplementary Fig. S4), suggesting an impairment in their pattern-separation ability, for which the DG and CA3 regions are necessary^26–28^. Thus, these behavioral data seem to be consistent with electrophysiological data showing impaired synaptic transmission and plasticity in the hippocampus.

These results showed that hippocampus-dependent memory formation is impaired in *Caps1* cKO mice, suggesting that CAPS1 plays a pivotal role in both STM and LTM formation in adult hippocampal circuits.

### CAPS1 is directly involved in hippocampus-associated learning abilities

**Figure 7:**
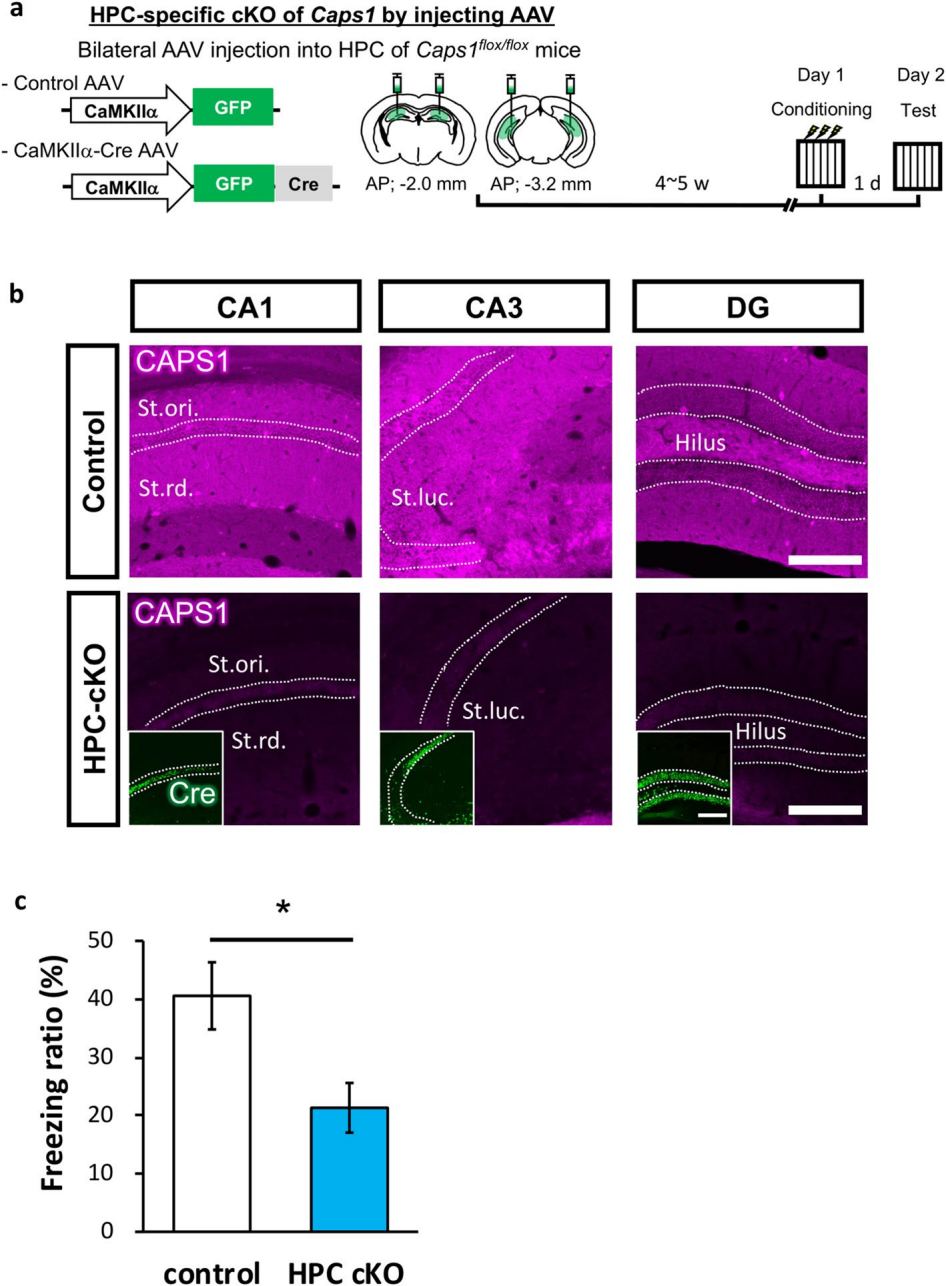
Memory deficits in adult mice with hippocampus-specific *Caps1* cKO. (**a**) The experimental scheme used for the knockout of *Caps1* in the adult hippocampus (HPC-cKO) and for testing CFC memory formation. (**b**) Representative images of immunohistochemical staining of CAPS1 in AAV-injected mice. The expression levels of the CAPS1 protein were sufficiently reduced in the hippocampus of HPC-cKO mice. The insets in the images of HPC-cKO slices show the expression of the Cre protein in the hippocampus. Scale bars, 200 µm. St.rd.: Stratum radiatum, St.ori.: Stratum oriens, St.luc: Stratum lucidum. (**c**) Mean freezing ratio during the CFC-LTM retrieval test. Control: n = 6 animals; HPC-cKO: n = 8 animals. *P* = 0.018, Student’s *t*-test.

The *Caps1* cKO mice used in our experiments lacked the *Caps1* gene during their early developmental stage (the *Emx1* promoter was driven until P8^29^). To exclude the possibility that the memory impairments observed in *Caps1* cKO mice were caused by potential secondary deficit(s) produced during hippocampal development, the *Caps1* gene was specifically knocked out in the adult hippocampus (HPC-cKO). Cre recombinase was expressed bilaterally in the dorsal and ventral hippocampi of *Caps1*^*fl/fl*^ mice using the AAV (CaMKIIα-Cre-AAV: AAV carrying Cre recombinase driven by the CaMKIIα promoter; Fig. 7a). Next, we confirmed that the levels of CAPS1 protein were significantly decreased in the CaMKIIα-Cre-AAV-infused hippocampus compared with those in the control-AAV-infused hippocampus at 4–5 weeks after AAV infusion (Fig. 7b). The mice were then trained in the CFC task and tested 1 d after training. HPC-cKO mice showed significantly decreased freezing ratios compared to those in the control mice (Fig. 7c). This result suggests that CAPS1 is directly involved in hippocampus-associated learning abilities, without remarkable developmental dysfunctions.

## Discussion

The CAPS1 protein, which was originally identified as a regulator of DCV exocytosis in neuroendocrine cells^8^, has recently been identified as an important SV exocytosis regulator in neurons^6,13,14^ and a candidate genetic risk factor in several psychiatric disorders, such as cognitive impairment and autism^30–32^. However, the functional significance of CAPS1 in mature neural circuits and brain function remains elusive. In this study, we used forebrain-specific *Caps1* cKO mice to clarify the role of CAPS1 in LTP in two distinct hippocampal circuits and its impact on brain function. *Caps1* cKO mice did not show robust changes in the expression levels of major exocytosis-regulator proteins or in developmental presynaptic formation. This was in agreement with previous reports in which the deletion of presynaptic proteins did not cause severe changes in molecular expression patterns or synaptic morphology^15,33,34^. These results support our hypothesis that CAPS1 is directly responsible for excitatory synaptic plasticity and learning abilities.

We demonstrated that TBS-induced LTP was induced and sustained, but extraordinary in the CA1 region of *Caps1* cKO slices. Considering the fact that hippocampal CA1 LTP is primarily a result of the activation of postsynaptic NMDA-type glutamate receptors^35^, we assumed that, in *Caps1* cKO slices, postsynaptic CA1 pyramidal cells retain the ability to evoke postsynaptic LTP, despite the extreme reduction in fEPSP amplitude. However, repeated application of TBS trains enhanced the potentiation ratio in *Caps1* cKO slices, while LTP was saturated in control slices. These findings suggest that postsynaptic LTP is not efficiently induced in *Caps1* cKO mice because of decreased presynaptic releasability, which can be attributed to the small size of the releasable pool at these synapses^13,14^.

In the CA3 region, LTP was severely impaired, likely because of differences in the mechanism of LTP induction between the CA1 and CA3 regions. CA3 LTP does not require postsynaptic NMDA receptor activation; rather, it warrants an increase in presynaptic release probability^36^. Assuming that CAPS1 plays an essential role in increasing presynaptic release probability, the induction of presynaptic LTP in the mossy fiber terminals may be seriously impaired in the absence of CAPS1. In accordance with this assumption, our electron microscopic data demonstrated that SVs are pushed away from the active zones in the CA3 presynapses of *Caps1* cKO mice, suggesting that the SV release probability is partially limited in these synapses. One limitation of our electron microscopic data is that the single-section-based information is too poor to cover more detailed ultramicrostructures, such as total SVs and whole presynaptic morphology, contrary to our previous study^14^. However, we believe that we have collected sufficient sample numbers to analyze docked SVs. Another point is that presynapses may be excited due to a lack of oxygen during perfusion and fixation. We consider that this problem is mostly compensated by recycling mechanisms of released SVs; therefore, the underestimation of docked SVs is not critical in our data.

In this study, we also showed that the specific abilities of hippocampus-dependent learning and memory were severely impaired in *Caps1* cKO mice. We verified a similar memory-defective phenotype in the HPC-cKO mice. These results indicate that the loss of CAPS1 in the hippocampus, but not in other forebrain regions, causes a deficiency in learning and memory. Conversely, *Caps1* cKO mice did not show impairments in the AFC-LTM test, in which hippocampal function is thought to be dispensable^37^. We assumed that impairments in learning ability are attributed to both CA1 and CA3 abnormalities. In CA1, not only the abnormal LTP magnitude, but also the low level of basal synaptic transmission is involved in the behavioral results. Conversely, the severe impairment of CA3 LTP induction should affect the learning performance, because dentate gyrus and CA3 neurons are required for CFC learning and memory^38–41^. In addition, CAPS1 deficits in axonal terminals may reduce synaptic input from the hippocampus to other brain areas such as the entorhinal cortex, which may also contribute to learning impairments.

However, the nature of the direct relationship between physiological impairments and memory deficits remains unclear.

**Figure 8:**
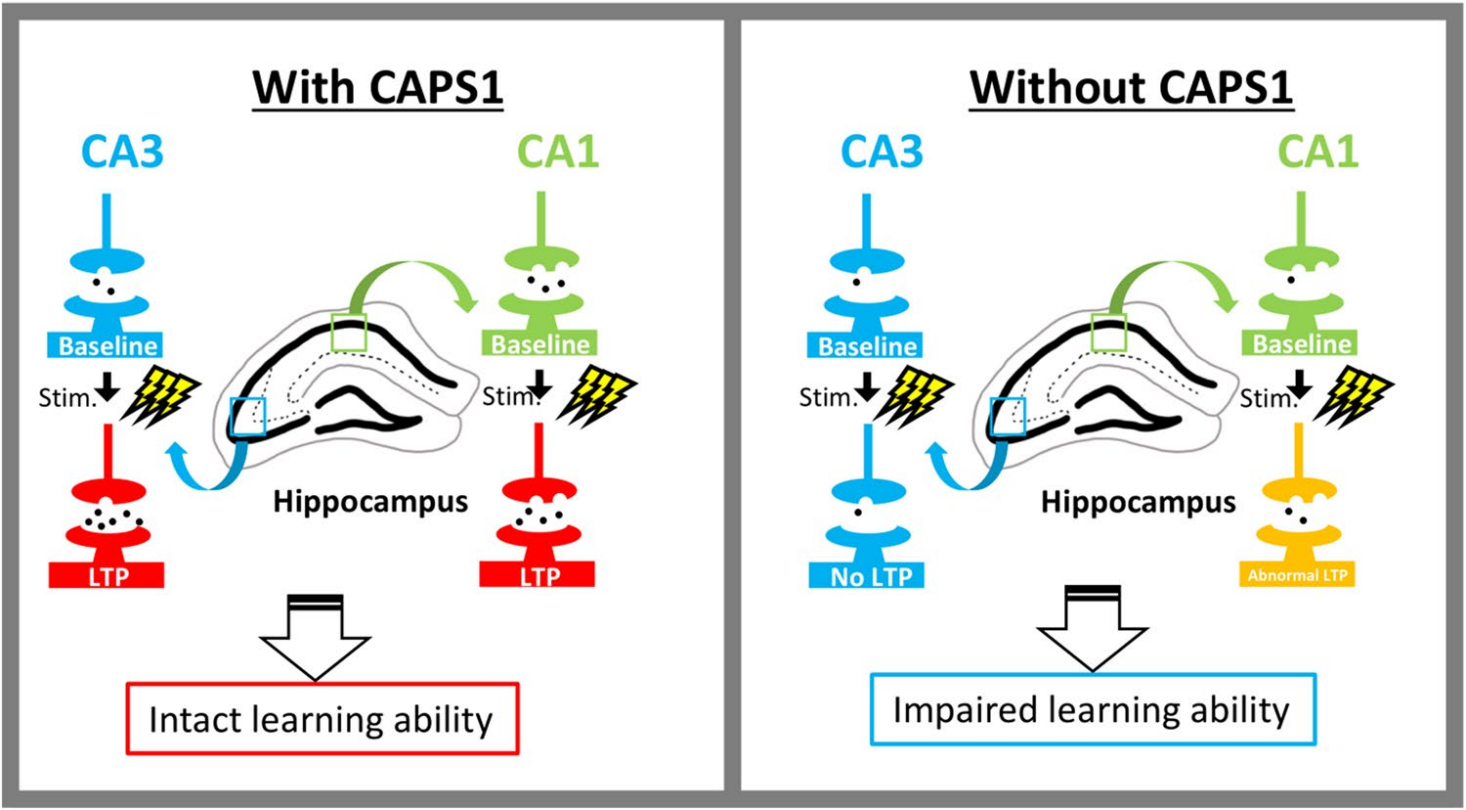
Schematic illustration of the role of CAPS1 in the hippocampal neural circuit and learning ability. Left: Physiological characteristics of the hippocampal neural circuits (CA3 and CA1 regions) with CAPS1. SVs are ready for exocytosis because CAPS1 renders SVs “docked” state, resulting in full LTP induced by TBS in both the CA3 and CA1 regions. This physiological plasticity is required for an intact learning ability. Right: Physiological characteristics of the hippocampal neural circuits (CA3 and CA1 regions) without CAPS1. SVs are to be away from the active zones because the absence of CAPS1 is insufficient to render SVs “docked” state, resulting in no or insufficient LTP induced by TBS in CA3 or CA1 regions, respectively. These physiological abnormalities underlie impaired learning ability.

In conclusion, we demonstrated for the first time the functional significance of CAPS1 not only in the neural circuit basis of the hippocampus, but also in hippocampus-associated learning behaviors. The CAPS1 protein appears to play a critical role in the appropriate regulation of CA1 LTP magnitude, as well as in the initiation of CA3 LTP in mature hippocampal circuits. These physiologically significant functions in both CA1 and CA3 regions ensure hippocampus-associated learning abilities and are possibly related to psychiatric disorders, including schizophrenia and autism, as some patients with these diseases reportedly exhibit differences in CAPS1 expression (Fig. 8).

## Supplementary Information

Supplementary Information

## Data Availability

The raw data supporting the conclusions of this article will be made available by the authors, without undue reservation.

## Abbreviations

AAV: Adeno-associated virus
AFC: Auditory fear-conditioning
CAPS1: Calcium-dependent activator protein for secretion 1
CFC: Contextual fear-conditioning
cKO: Conditional knock out
CNS: Central nervous system
DCV: Dense-core vesicle
DG: Dentate gyrus
fEPSP: Field excitatory postsynaptic potential
HPC-cKO: Hippocampus-specific conditional knockout
LTM: Long-term memory
LTP: Long-term potentiation
STM: Short-term potentiation
SV: Synaptic vesicle
TBS: Theta-burst stimulation
