# Supplementary material for: CAPS1 is involved in hippocampal synaptic plasticity and hippocampus-associated learning
Source: Sci Rep. 2021 Apr 21;11:8656. doi: 10.1038/s41598-021-88009-w (PMC8060421; doi:10.1038/s41598-021-88009-w)
Supplement: Supplementary file 1 — Supplementary Information [file 41598_2021_88009_MOESM1_ESM.pdf]

Supplementary Information

CAPS1 is involved in hippocampal synaptic plasticity and hippocampus-associated learning

Chiaki Ishii[1], Natsumi Shibano[1], Mio Yamazaki[1], Tomoki Arima[1], Yuna Kato[1], Yuki Ishii[1], Yo Shinoda[2], Yugo Fukazawa[3], Tetsushi Sadakata[4], Yoshitake Sano[1] and Teiichi Furuichi[1*]

1. Department of Applied Biological Science, Faculty of Science and Technology, Tokyo University of Science, 2641 Yamazaki, Noda, Chiba 278-8510, Japan
2. Department of Environmental Health, School of Pharmacy, Tokyo University of Pharmacy and Life Sciences, 1432-1 Horinouchi, Hachioji, Tokyo 192-0392, Japan
3. Department of Brain Structure and Function, Faculty of Medical Sciences, University of Fukui, Yoshida-gun, Fukui 910-1193, Japan.
4. Education and Research Support Center, Gunma University Graduate School of Medicine, Maebashi, Gunma 371-8511, Japan

*** Corresponding author**
E-mail: tfuruichi@rs.tus.ac.jp (TF)

a

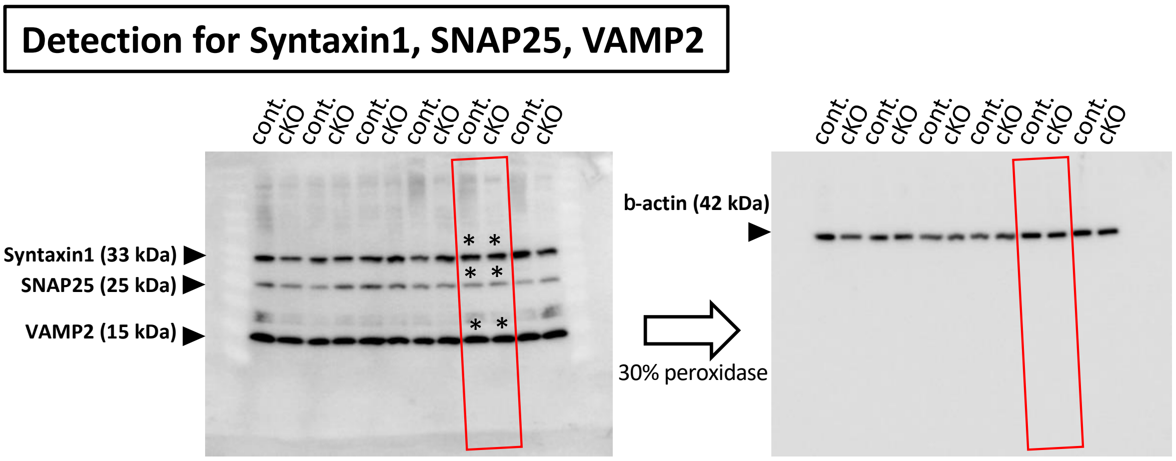

b

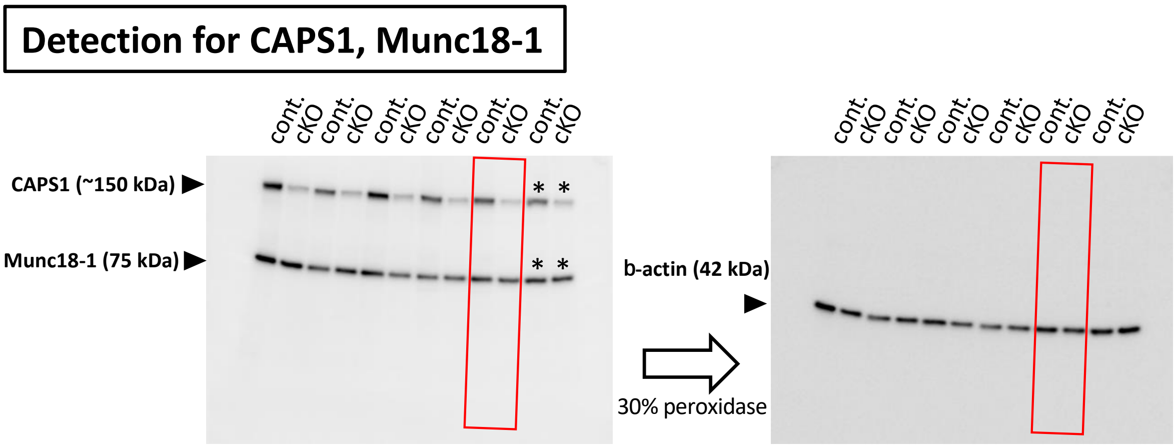

Figure S1. Whole membranes used for quantitative analyses of Western blotting.
(**a**) Whole membranes were used for the detection of syntaxin1, SNAP25, and synaptobrevin/VAMP2 (Left); β-actin was used as the internal control (right). All targeted proteins were detected on the same membrane. Each targeted band was identified based on the band size. The sizes of the marker bands are indicated the left of the membrane. β-actin was detected on the same membrane after treatment with 15% peroxidase to erase the previously targeted bands. The red frame indicates each representative paire of lanes depicted in Fig.1-b. The astarisk avobe the band indicates each representative band depicted in Fig.1-c.
(**b**) Whole membranes were used for the detection of CAPS1 and Munc18-1 (Left); β-actin was used as the internal control (right). The targeted band was identified based on the band size. The sizes of the marker bands are indicated to the left of the membrane. β-actin was detected on the same membrane after treatment with 15% peroxidase to erase the previously targeted bands. The red frame indicates each representative paire of lanes depicted in Fig.1-a. The astarisk avobe the band indicates each representative band depicted in Fig.1-c.

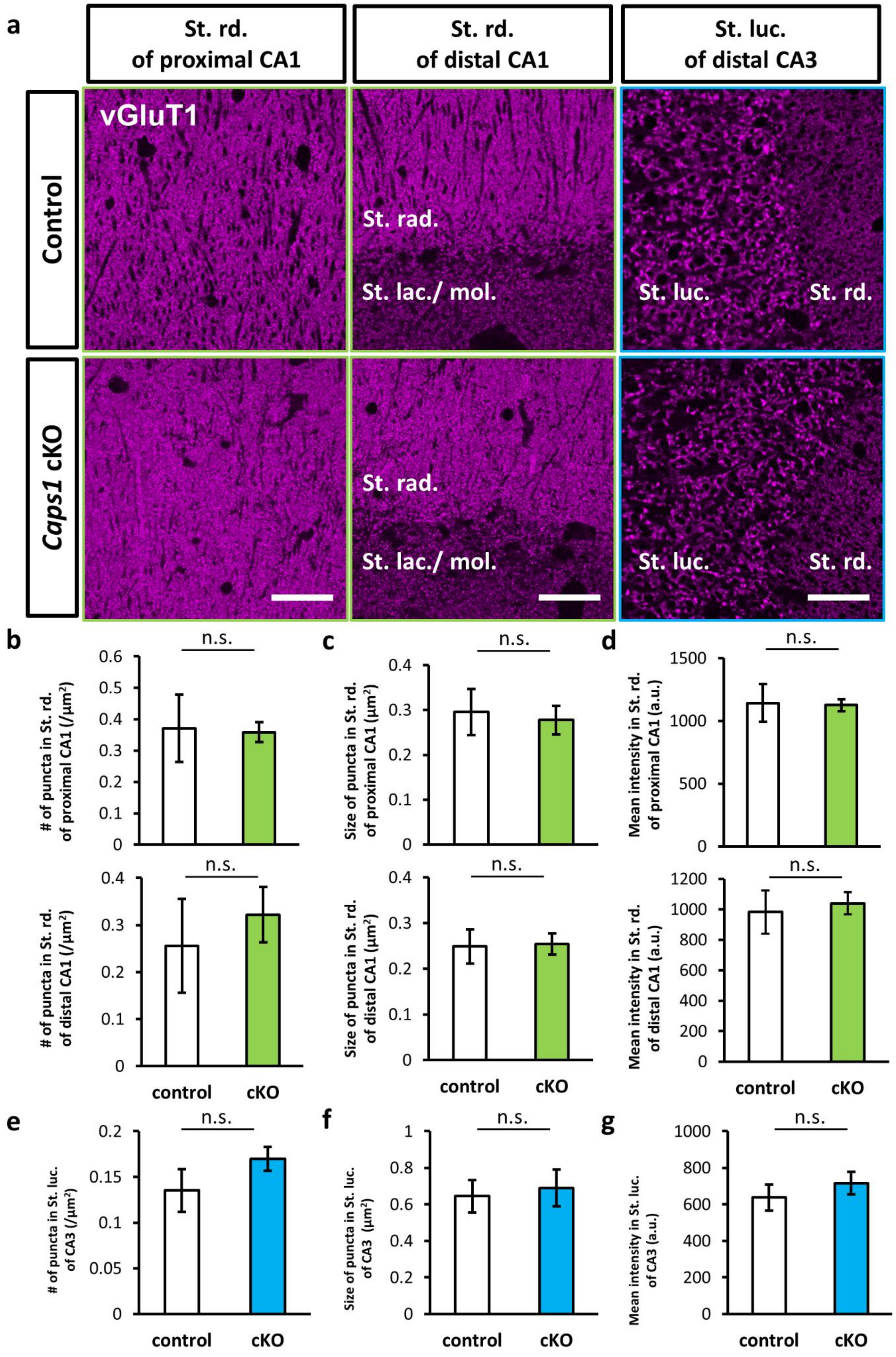
a
St. rd. of proximal CA1
St. rd. of distal CA1
St. luc. of distal CA3
Control
Caps1 cKO
vGluT1
St. rad.
St. lac./ mol.
St. luc.
St. rd.
b
c
d
e
f
g
n.s.
# of puncta in St. rd. of proximal CA1 (/μm²)
Size of puncta in St. rd. of proximal CA1 (μm²)
Mean intensity in St. rd. of proximal CA1 (a.u.)
# of puncta in St. rd. of distal CA1 (/μm²)
Size of puncta in St. rd. of distal CA1 (μm²)
Mean intensity in St. rd. of distal CA1 (a.u.)
# of puncta in St. luc. of CA3 (/μm²)
Size of puncta in St. luc. of CA3 (μm²)
Mean intensity in St. luc. of CA3 (a.u.)
control
cKO

Supplementary Fig. S2. Presynaptic formation in *Caps1* cKO hippocampus

(**a**) Representative images of the immunohistochemical staining of vGluT1 in the Schaffer collateral and mossy fiber terminal area. Expression pattern of vGluT1 in control (upper panels) and cKO (lower panels) mice. Scale bars, 30 µm. St. rd., stratum radiatum; St. luc., stratum lucidum; St. lac./mol., stratum lacunosum/moleculare. (**b**) Number of vGluT1-positive puncta in the stratum radiatum of the CA1 region (upper, proximal; lower, distal). Control, n = 4 animals; *Caps1* cKO, n = 5 animals in the proximal region. *P* = 0.902, Student's *t*-test (upper). Control, n = 4 animals; *Caps1* cKO, n = 5 animals in the distal region (*P* = 0.567, Student's *t*-test (lower)). (**c**) Average size of a single vGluT1-positive punctum in the stratum radiatum of the CA1 region (upper, proximal; lower, distal). Control, n = 4 animals; *Caps1* cKO, n = 5 animals in the proximal region (*P* = 0.771, Student's *t*-test (upper)). Control, n = 4 animals; *Caps1* cKO, n = 5 animals in the distal region (*P* = 0.905, Student's *t*-test (lower)). (**d**) Mean intensity of the vGluT1 signal in the stratum radiatum of the CA1 region (upper, proximal; lower, distal). Control, n = 4 animals; *Caps1* cKO, n = 5 animals in the proximal region (*P* = 0.920, Student's *t*-test (upper)). Control, n = 4 animals; *Caps1* cKO, n = 5 animals in the distal region (*P* = 0.720, Student's *t*-test (lower)). (**e**) Number of vGluT1-positive puncta in the stratum lucidum of the CA3 region. Control, n = 4 animals; *Caps1* cKO, n = 5 animals (*P* = 0.216, Student's *t*-test). (**f**) Average size of a single vGluT1-positive punctum in the stratum lucidum of the CA3 region. Control, n = 4 animals; *Caps1* cKO, n = 5 animals (*P* = 0.753, Student's *t*-test). (**g**) Mean intensity of the vGluT1 signal in the stratum lucidum of the CA3 area. Control, n = 4 animals; *Caps1* cKO, n = 5 animals for the distal region (*P* = 0.428, Student's *t*-test).

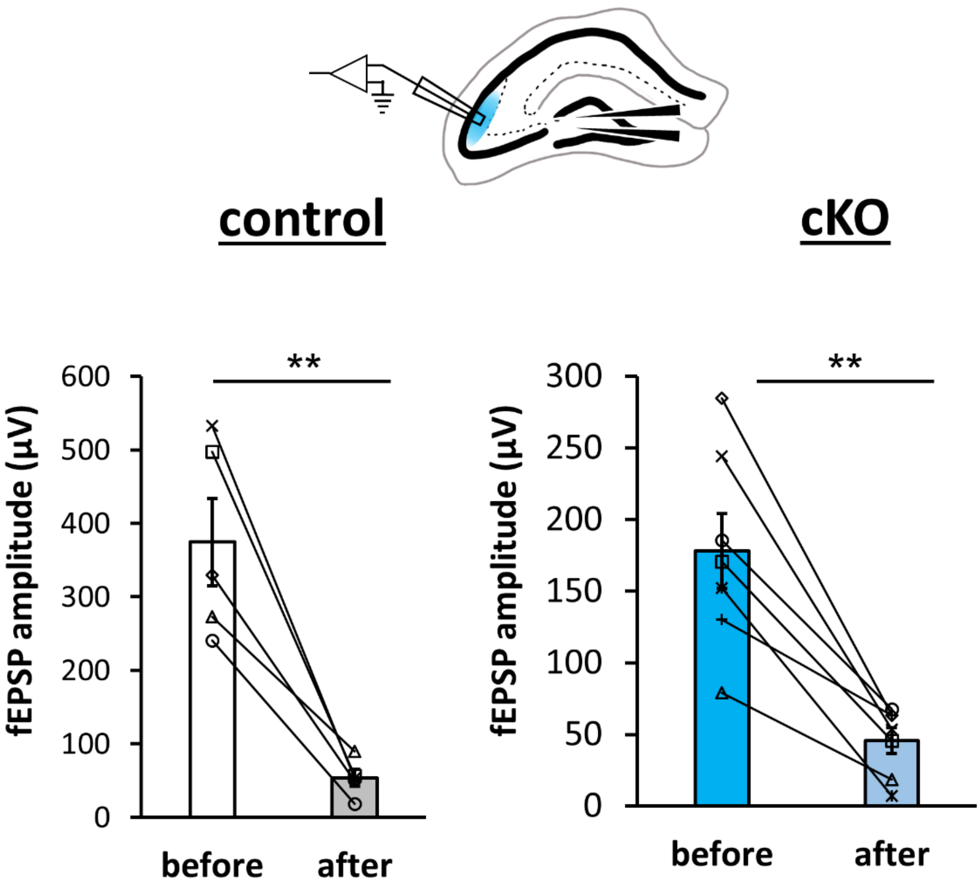

Supplementary Fig. S3. Changes in fEPSP amplitude at DG-CA3 synapses after the administration of DCG4

DCG4, an agonist of the type II metabotropic glutamate receptors, was added into the electrophysiological recording chamber to block the mossy fiber input from dentate gyrus. The reduction of fEPSP after DCG4 input indicates that the recorded fEPSP is mostly attributed to mossy fiber input. fEPSP amplitudes at DG-CA3 synapses of control and cKO before and after the administration of DCG4 are shown. Control, n = 5 animals (P = 0.006, paired t-test); *Caps1* cKO, n = 7 animals (P = 0.001, paired t-test).

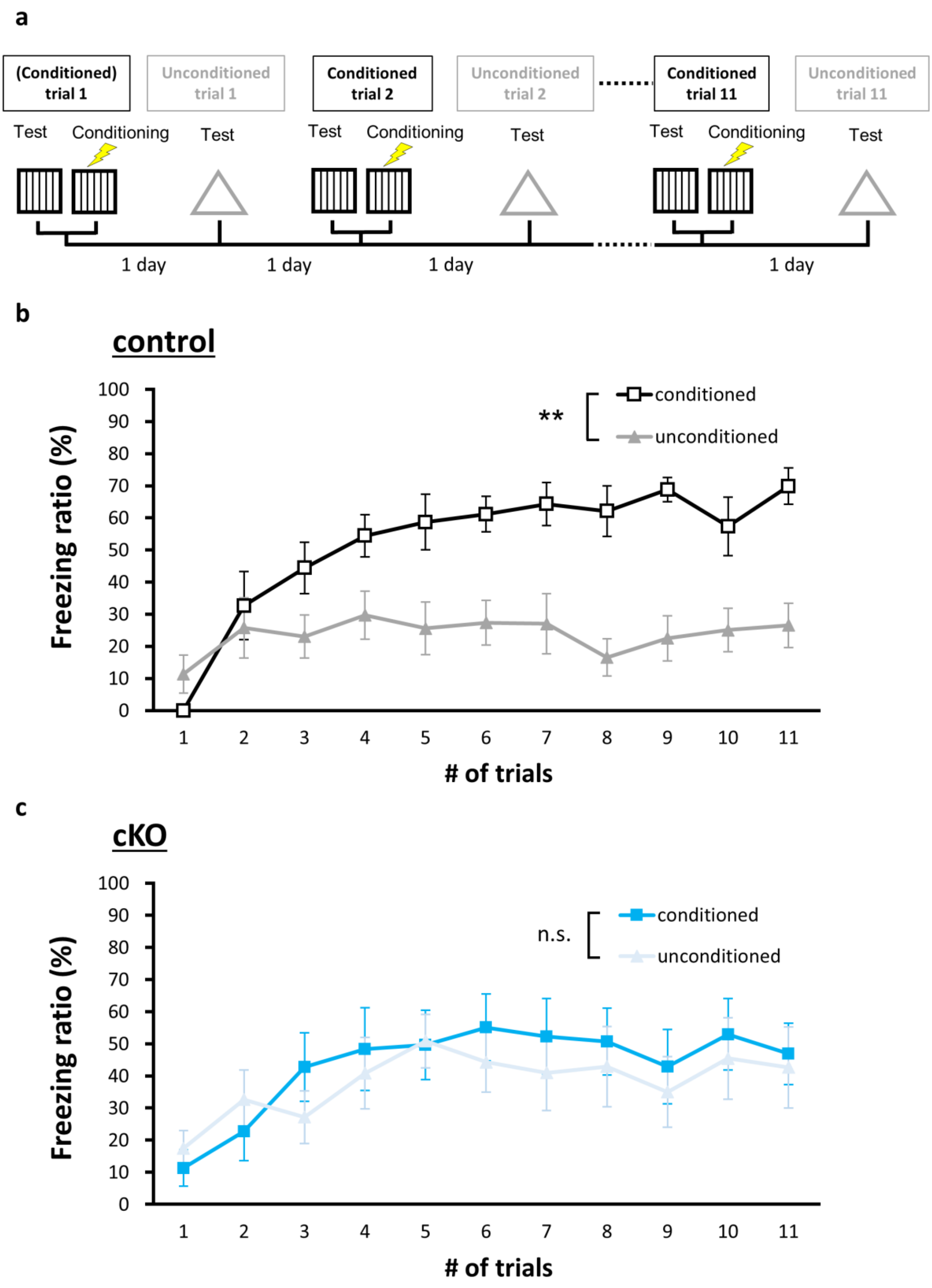

Supplementary Fig. S4. Impairment of the context-discrimination task in *Caps1* cKO mice

(**a**) Experimental scheme used in the context-discrimination task. Conditioned trials and unconditioned trials were conducted alternatively every other day. (**b**) Changes in the

freezing ratio during the context-discrimination task in control mice. Conditioned trials: n = 8 animals, 11 trials; unconditioned trials: n = 8 animals, 11 trials. **$P < 0.01$, repeated two-way ANOVA (Context; $F_{(1, 14)} = 12.7$, $P = 0.003$). (**c**) Changes in the freezing ratio during the context-discrimination task in *Caps1* cKO mice. Conditioned trials: n = 8 animals, 11 trials; unconditioned trials: n = 8 animals, 11 trials. No significant change between the two trials was found, repeated two-way ANOVA (Context; $F_{(1, 14)} = 0.19$, $P = 0.668$).

Fig. #	contents	Test type	# of group (factor)	p value	Power	# of samles
1-c	Western blotting (CAPS1)	Student's t-test	2	<0.001	0.985	6:6
2-b	Mean intensity (Syntaxin1; CA3-CA1)	Student's t-test	2	0.001	0.997	5:5
3-b	Tne number of docked SVs	Student's t-test	2	0.028	1.000	71:75
4-b	% of slope right after TBS (CA3-CA1)	Student's t-test	2	0.014	0.969	6:5
4-h	Repeated TBS (CA3-CA1)					
	control; 4th vs cKO; 4th	Student's t-test	2	0.038	0.743	5:5
	control; 5th vs cKO; 5th	Student's t-test	2	0.005	0.979	5:5
	control; 6th vs cKO; 6th	Student's t-test	2	0.015	0.893	5:5
	cKO; 1st vs cKO; 4th	Student's t-test	2	0.007	0.871	5:5
	cKO; 1st vs cKO; 5th	Student's t-test	2	0.004	0.989	5:5
	cKO; 1st vs cKO; 6th	Student's t-test	2	0.022	0.851	5:5
5-a	IO curve (DG-CA3)	Repeated two-way ANOVA	2	<0.001		5:7
5-b	PPF (DG-CA3)	Repeated two-way ANOVA	2	<0.001		5:7
5-e	% of slope 2h after TBS (DG-CA3)	Student's t-test	2	0.023	0.973	5:7
5-g	Repeated TBS (DG-CA3)					
	control; 1st vs cKO; 1st	Student's t-test	2	0.032	0.840	6:5
	control; 4th vs cKO; 4th	Student's t-test	2	0.036	0.753	6:5
	control; 5th vs cKO; 5th	Student's t-test	2	0.034	0.818	6:5
	control; 6th vs cKO; 6th	Student's t-test	2	0.027	0.830	6:5
	control; 1th vs control; 3rd	Student's t-test	2	0.019	0.867	6:6
	control; 1th vs control; 4th	Student's t-test	2	0.036	0.759	6:6
	control; 1th vs control; 5th	Student's t-test	2	0.028	0.801	6:6
	control; 1th vs control; 6th	Student's t-test	2	0.041	0.709	6:6
6-a	Changes of freezing ratio during CFC test (LTM)	Repeated two-way ANOVA	2	<0.001		6:5
6-b	Total freezing ratio (LTM)	Student's t-test	2	<0.001	0.999	6:5
6-d	Changes of freezing ratio during CFC test (STM)	Repeated two-way ANOVA	2	<0.001		19:11
6-f	Total freezing ratio (STM)	Student's t-test	2	<0.001	0.930	19:11
7-c	Total freezing ratio (STM; control vs HIP-cKO)	Student's t-test	2	0.018	0.713	6:8

Supplementary Table S1. Summary of statistical parameters
Statistical parameters, inclusing p values and power calucated retrospectively, are shown in this study, only for the quantitative data with statsistical significances.
